# Case Report: A case of Culler-Jones syndrome caused by *GLI2* gene mutation

**DOI:** 10.3389/fped.2025.1699082

**Published:** 2025-12-19

**Authors:** Xiaomei Xie, Youfen Wei, Ye Li, Junyan Wang, Yating Zhang, Jie Wu, Fan Wang

**Affiliations:** Department of Neonatology, The Second Hospital of Lanzhou University, Lanzhou, China

**Keywords:** Culler-Jones syndrome, developmental delay, epilepsy, *GLI2*, polydactyly, whole exome sequencing

## Abstract

Culler-Jones syndrome (CJS) (OMIM: 615849) is a rare genetic condition characterized by considerable phenotypic variability. This case reports a 5-day-old male neonate who presented with postaxial polydactyly, growth restriction, and recurrent epileptic seizures. A thorough clinical workup, including laboratory investigations, imaging, and genetic analysis, resulted in a confirmed diagnosis of Culler-Jones syndrome. Peripheral blood samples collected from the proband and his parents were used for DNA extraction. Whole-exome sequencing (WES) identified a heterozygous nonsense variant in the *GLI2* gene, [c.2137(exon13)G>T/p.(E713,857) (NM_001374353)], which was subsequently validated by Sanger sequencing and determined to be maternally inherited. This mutation has not been previously documented in the literature. By detailing the clinical presentation, genetic findings, and relevant context, this case report aims to broaden the known phenotypic spectrum of Culler-Jones syndrome and support clinicians in early detection and diagnosis.

## 1 Introduction

Culler-Jones syndrome (CJS; OMIM: 615849) is a rare autosomal-dominant disorder caused by pathogenic variants in the *GLI2* gene on chromosome 2q14 (1). The syndrome is characterized by incomplete penetrance and variable expressivity, resulting in significant clinical heterogeneity and frequent diagnostic uncertainty. Core clinical features include hypopituitarism, postaxial polydactyly, seizures, intellectual disability, and growth impairment. Other manifestations described in some patients include craniofacial abnormalities, such as midfacial hypoplasia, cleft lip or palate, ptosis, and microcephaly, as well as genital anomalies like micropenis and cryptorchidism (2, 3). Recent case reports from various regions have continued to broaden the recognized phenotypic spectrum of the disorder, yet many aspects of CJS remain insufficiently defined. Interpretation of *GLI2* variants is further complicated by the presence of certain mutations in the general population that lack clear clinical consequences, as well as by environmental factors that influence phenotypic expression (4–6). The heterozygous *GLI2* variant identified in our patient [c.2137(exon13)G>T/p.(E713,857), NM_001374353] has not been previously documented. This case contributes to the expanding clinical and genetic understanding of CJS and underscores the value of genetic testing for accurate diagnosis and early intervention.

## 2 Case report

The proband, a 5-day-old male neonate, was admitted to the Second Hospital of Lanzhou University in October 2024 with a two-day history of generalized jaundice affecting the skin and mucous membranes. At the time of admission, the mean transcutaneous bilirubin level measured in the obstetrics department was 315 μmol/L. He was subsequently referred to the neonatology unit and hospitalized with a preliminary diagnosis of neonatal hyperbilirubinemia.

He was the second child of healthy, non-consanguineous parents. Delivery occurred via cesarean section at 37 + 3 weeks of gestation, and his birth weight was 2.63 kg. Polydactyly of all four extremities was noted at birth. There was no history of asphyxia, cyanosis, or need for resuscitation. The mother, who was 145 cm tall, also had bilateral postaxial hexadactyly. She reported no gestational diabetes and denied exposure to teratogens, toxins, or radiation during pregnancy. The patient's older sibling had died at 6 months of age due to congenital heart disease and pneumonia (further details unavailable). The maternal aunt had experienced multiple adverse pregnancy outcomes, including two miscarriages, and several maternal relatives also had polydactyly. The father was clinically healthy and 173 cm tall.

**Figure 1:**
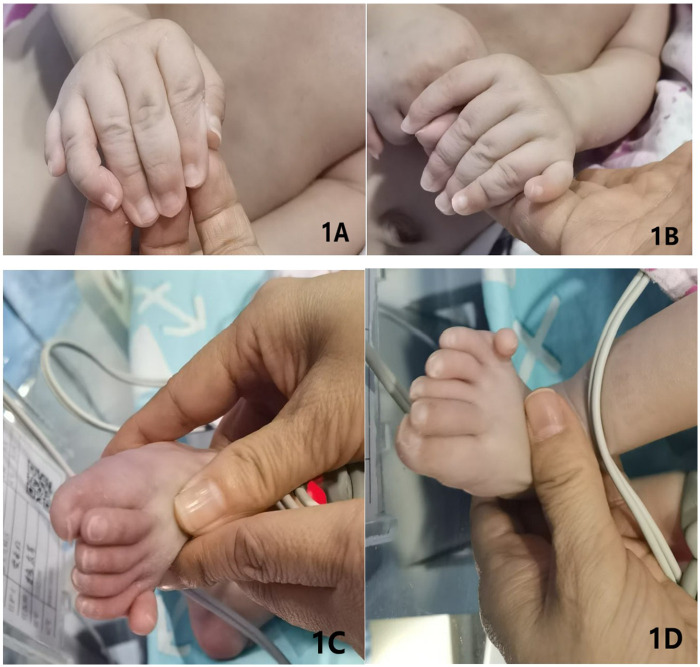
**(A–D)** Show the child's right hand, left hand, left foot, and right foot in sequence, all of which exhibited hexadactyly (six fingers/toes).

On admission, physical examination revealed: temperature 36.7 °C, heart rate 140 bpm, respiratory rate 35 breaths/min, and SpO₂ 95%. Anthropometric measurements included length 46 cm, head circumference 34 cm, chest circumference 32 cm, and weight 2.49 kg. The infant was alert and appropriately responsive. Significant findings included diffuse jaundice, scleral icterus, and normal primitive reflexes without pathological signs. Limb tone was normal. Postaxial polydactyly was present in all four limbs, with a total of 24 digits (Figures 1A–D).

Laboratory and imaging assessments showed unremarkable results for complete blood count, arterial blood gas analysis, chest radiography, echocardiography, and abdominal ultrasonography (liver, gallbladder, pancreas, spleen). Liver function testing revealed total bilirubin 254.3 μmol/L, direct bilirubin 13.7 μmol/L, and indirect bilirubin 240.6 μmol/L (Table 1). Cranial ultrasound was normal. Symptomatic management was initiated, including thermal support, adequate feeding, and phototherapy. On the second day of hospitalization at 14:37, the patient experienced an episode of bradycardia with a heart rate below 60 bpm, accompanied by profound oxygen desaturation. Immediate resuscitation was initiated, and by 14:39, cardiac activity had stabilized at 105–115 bpm, with oxygen saturation improving to 93%–95%. Mechanical ventilation was commenced thereafter. Following resuscitation, the infant demonstrated weak spontaneous breathing, sluggish pupillary reactions, reduced responsiveness, involuntary limb tremors, and increased muscle tone. Despite treatment with phenobarbital and midazolam, seizure activity persisted.

**Table 1:** Laboratory test results of the child.

Parameter	Age (days)		Reference range
	7	31	
ACTH	10.0	–	7.2–63.3 pg/mL
Cortisol	15	–	4.8–19.5 µg/dL
Blood glucose	3.6	5.6	3.90–6.10 mmol/L
Total bilirubin	254.3	5.1	<21 µmol/L
Direct bilirubin	13.7	2.4	<4 µmol/L
Indirect bilirubin	240.6	2.7	0–22.1 µmol/L
GGT	68	37	9–150 U/L
Hemoglobin	165	138	97–183 g/L
AST	38	24	21–80 U/L
ALT	16	35	8–71 U/L
Na	134.5	135.6	135–150 mmol/L
LH	0.38	–	0–6 mIU/mL
PRL	75.5	–	2.1–17.7 ng/mL
Free T3	5.25	3.25	2.49–7.10 pmol/L
Free T4	14.01	11.78	12.00–29.34 pmol/L
TSH	8.067	1.74	0.47–12.25 pmol/L
Growth hormone	20.1	–	0–5.00 ng/mL
IGF-1	22.8	–	18–129.00 ng/mL
FSH	1.22	–	1.40–18.10 mIU/mL

ACTH, adrenocorticotropic hormone; TSH, thyroid stimulating hormone; AST, aspartate aminotransferase; ALT, alanine aminotransferase; IGF-1, insulin-like growth factor 1; GGT, γ-glutamyl transpeptidase; LH, luteinizing hormone; FSH, follicle-stimulating hormone; PRL, prolactin; –, not determined.

The child remained in critical condition throughout a prolonged hospitalization of more than 5 months, requiring multiple episodes of intubation and invasive mechanical ventilation, with no successful attempts at weaning. Neurologic function remained severely impaired, characterized by minimal responsiveness, recurrent seizures, hypertonia, and oromotor dysfunction. Because of an inability to suck or swallow, he required nasogastric feeding with preterm formula. Oral secretions were thick and abundant. Antiepileptic therapy, including levetiracetam and phenobarbital, provided only limited clinical improvement.

Serial laboratory evaluations, including haematology, infection markers, blood cultures, electrolytes, plasma ammonia, pituitary hormones (growth hormone, adrenocorticotropic hormone, thyroid-stimulating hormone), insulin-like growth factor-1, luteinizing hormone, follicle-stimulating hormone, prolactin, and cerebrospinal fluid analysis, were all within normal limits (Table 1).

**Figure 2:**
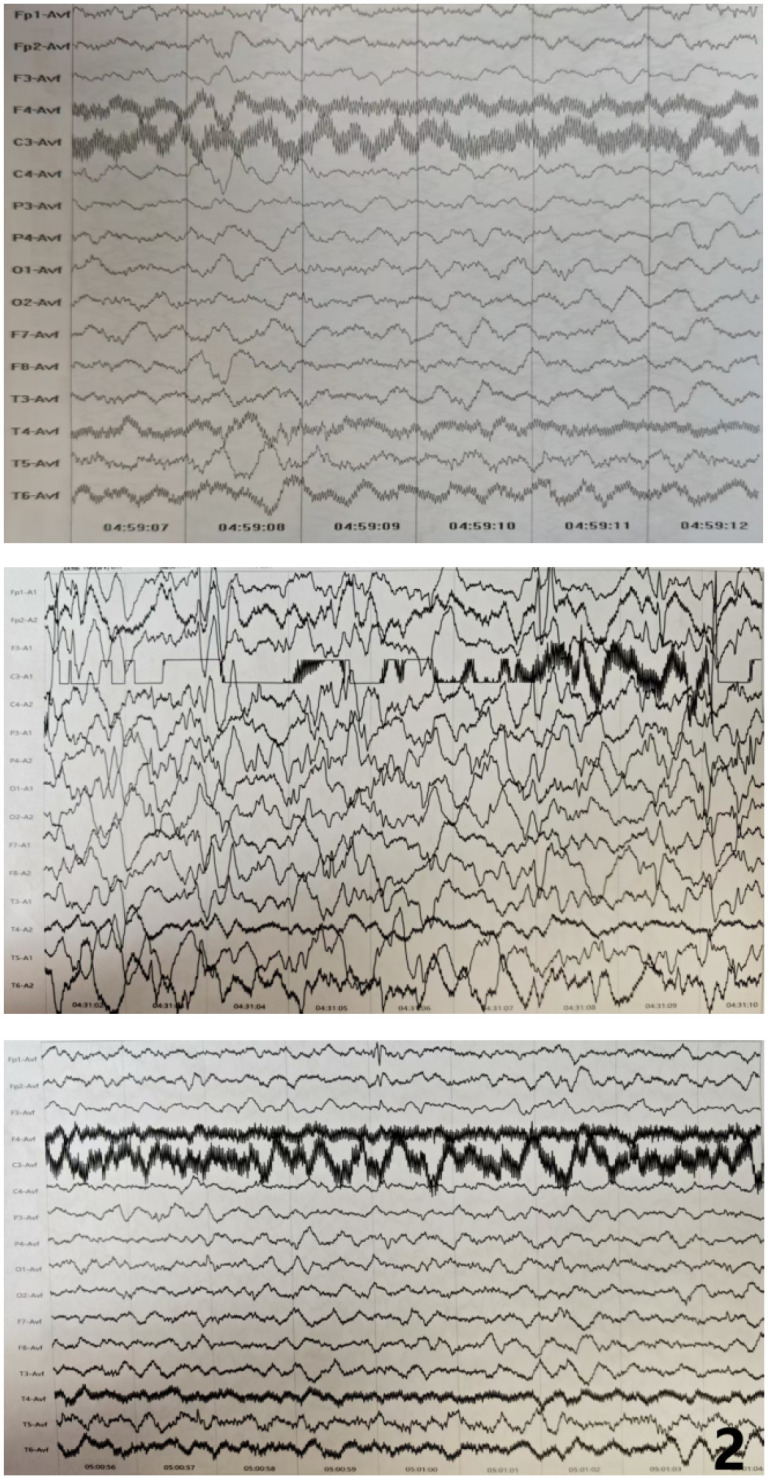
The 24-hour ambulatory electroencephalogram (EEG) for the child exhibiting abnormal findings. The background was composed of diffuse slow waves. During sleep, continuous discharges (approaching persistent discharges) of diffuse slow waves mixed with a large number of spike-slow waves, sharp waves, and sharp-slow waves were noted, occuring primarily in the bilateral anterior head regions, with right anterior predominance.

Electroencephalography (EEG) showed a background dominated by diffuse, low- to medium-amplitude 2–3 Hz delta activity, generally symmetric between hemispheres. These waves were not suppressed, and scattered low-amplitude fast waves were present across leads. Sleep architecture was poorly defined, with no clear sleep vertex waves or spindles, and the sleep–wake cycle was difficult to distinguish. During sleep, medium- to high-amplitude diffuse slow waves mixed with abundant spike-and-slow waves, spikes, and sharp-and-slow waves were observed, predominantly in the bilateral frontal and anterior temporal regions, with right-sided predominance. A smaller amount of epileptiform activity was also present during wakefulness. A 24-hour ambulatory EEG further demonstrated a diffuse slowing of the background. During sleep, nearly continuous discharges, including spike-and-slow waves, sharp waves, and sharp-and-slow waves, were concentrated in the bilateral anterior head regions, with greater prominence on the right (Figure 2).

**Figure 3:**
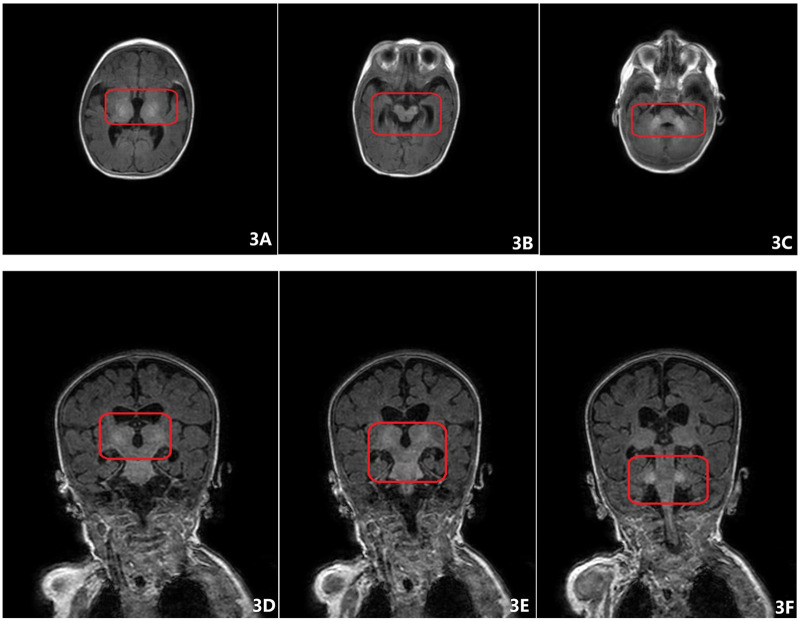
**(A–C)** T1-weighted axial images. **(A)** Symmetric slightly hyperintense signals on T1WI are noted in the bilateral basal ganglia and thalamus. **(B)** Symmetric slightly hyperintense signals on T1WI are observed in the cerebral peduncles. **(C)** Symmetric slightly hyperintense signals on T1WI are seen in the pontine brachia. **(D–F)** T1-weighted coronal images. Symmetric slightly hyperintense signals on T1WI in the aforementioned regions are similarly demonstrated.

Brain MRI revealed symmetric short T1 signals in the bilateral basal ganglia, thalamus, and cerebellar peduncles, along with T1 hyperintense signals in the bilateral dentate nuclei. The pituitary gland showed normal morphology and structure (Figures 3A–F). These symmetric abnormalities in deep brain structures suggest possible deposition of metabolites or minerals. However, evaluations for hereditary metabolic diseases, including mitochondrial encephalopathies and amino acid or organic acid metabolism disorders, were unremarkable.

**Figure 4:**
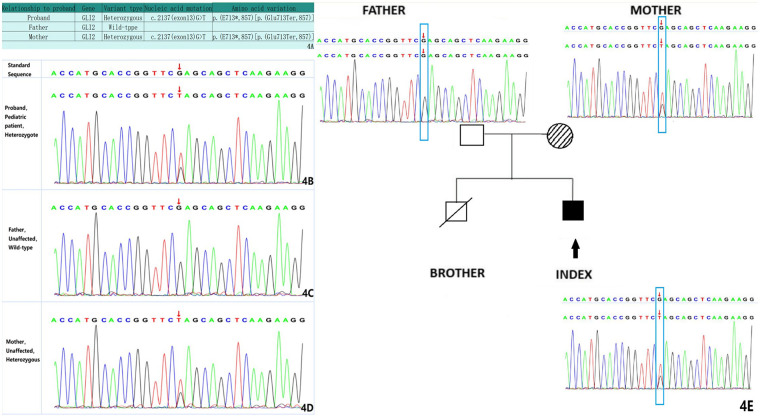
A table summarizing the GLI2 mutation types and mutation sites of the child and his parents **(A)**. The heterozygous c.2137 (exon13) G>T [p.(Glu713Ter,857)] mutation was identified in the child **(B)**. The forward sequencing result for the patient's father (WT) **(C)**. The forward sequencing result for the patient's mother, revealing the presence of the heterozygous c.2137 (exon13) G>T [p.(Glu713Ter,857)] mutation **(D)**. Family pedigree and electropherogram of heterozygous c.2137 (exon13) G>T [p.(Glu713Ter,857)] mutation in the GLI2 gene. Full-black filled box indicates index case with Culler-Jones syndrome phenotype, shaded boxes indicate mother who are also heterozygous for the identical mutation with incomplete phenotype, empty box indicates father with wild type, empty box with a diagonal line indicates the deceased elder brother **(E)**.

Whole-exome sequencing (Figures 4A–E) identified a heterozygous nonsense variant in *GLI2* [NM_001374353: c.2137(exon13)G>T/p.(E713,857)] located at chr2:121744085. Segregation analysis by Sanger sequencing confirmed maternal inheritance of the variant, while the father carried the wild-type allele. Integrating the clinical presentation, neuroimaging findings, and genetic results, the patient was diagnosed with Culler-Jones syndrome with severe neurological involvement. Given the poor prognosis and ongoing clinical decline, the family elected to discontinue intensive medical care, and the infant was discharged home.

## 3 Results

Based on the clinical presentation, ancillary examinations, and molecular findings, the patient was diagnosed with Culler-Jones syndrome, accompanied by epilepsy, developmental delay, and neonatal hyperbilirubinemia. Whole-exome sequencing revealed a heterozygous truncating variant in the *GLI2* gene: NM_001374353.1(*GLI2*): c.2137(exon13)G>T; p.(Glu713Ter,857). The *GLI2* gene is classified as haploinsufficient in the ClinGen database. This stop-gain variant was absent from major population reference databases, including the 1000 Genomes Project and gnomAD, indicating that it is extremely rare.

Using the American College of Medical Genetics and Genomics (ACMG) criteria (7), this variant meets the classification of likely pathogenic (LP), supported by: PVS1 (strong evidence; a nonsense mutation in a gene where loss of function is an established disease mechanism) and PM2 (supporting evidence; extremely low population frequency). Furthermore, the variant has not been reported in gnomAD, HGMD (Human Gene Mutation Database), or ClinVar.

At the molecular level, the substitution of thymine (T) for guanine (G) at nucleotide position 2,137 converts the codon for glutamic acid (Glu) at position 713 into a premature termination codon (Ter). This change introduces an early stop, leading to the truncation of the protein by 857 amino acids before its normal endpoint. The resultant truncated product would terminate polypeptide synthesis prematurely and disrupt normal GLI2 protein function. Segregation analysis confirmed that the variant was maternally inherited and absent in the father.

## 4 Discussion

The *GLI2* gene belongs to the gli-kruppel family of transcription factors and was first identified through its amplification in human gliomas (8). Located on chromosome 2q14, *GLI2* comprises 14 exons and encodes a protein of 1,586 amino acids (1). The GLI2 protein contains a zinc-finger DNA-binding domain and functions as a transcription factor, demonstrating repressive activity at its N-terminus and activating activity at its C-terminus. Depending on species-specific and cellular contexts, *GLI2* can promote or inhibit transcription and, together with *GLI1*, *GLI3*, and *Shh*, regulates downstream gene expression within the Hedgehog (Hh) signaling pathway. The Hh pathway plays a critical role in the development of the central nervous system, distal limb formation, and the pathogenesis of various tumors (9).

Despite its biological significance, *GLI2* is a large and highly polymorphic gene. Functionally impactful variants have been reported in both symptomatic and asymptomatic individuals, reflecting the incomplete penetrance of the condition (8). The considerable phenotypic variability among *GLI2* mutation carriers is likely influenced by genetic background, epigenetic regulation, and modifier genes within the Sonic Hedgehog (SHH) pathway, including *SHH*, *ZIC2*, *SIX3*, *PTCH1*, *GLI3*, and *TGIF* (10–13). Clinical manifestations associated with *GLI2* mutations range widely, from individuals with no apparent abnormalities to those presenting with polydactyly, craniofacial dysmorphisms, pituitary defects, Culler-Jones syndrome, and holoprosencephaly type 9 (HPE9; OMIM: 610829) (2, 14). The overall prevalence of *GLI2*-related disorders is estimated to be fewer than 1 per 1,000,000 individuals (6).

CJS is most commonly associated with pituitary hormone deficiencies, ectopic posterior pituitary, and postaxial polydactyly. However, other manifestations such as growth retardation, seizures, hypoglycemia, and midline structural abnormalities have also been reported (15). Database searches indicated that the variant identified in this patient represents a previously unreported *GLI2* mutation (1–4, 10–12, 14–23). This truncating mutation involves a guanine-to-thymine substitution at nucleotide position 2,137 in exon 13 of the *GLI2* coding region. This single-nucleotide change converts the codon for glutamic acid (Glu) at position 713 into a premature stop codon (Ter), generating a truncated protein lacking the final 857 amino acids of the normal sequence.

**Figure 5:**
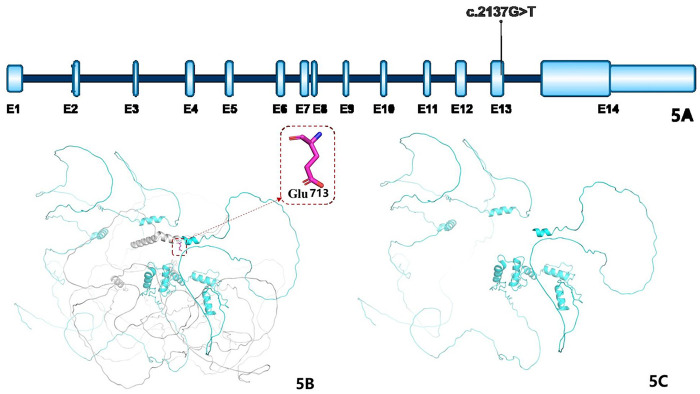
**(A)** Shows the structural diagram of the GLI2 gene: the blue parts represent exons (abbreviated as E), and the teal parts represent introns; the mRNA variant number is NM_001374353.1, which consists of 14 exons, and the marked variant sites are the cases in this study. **(B)** Shows the schematic diagram of the three-dimensional structure of the wild-type GLI2 protein (the gray part represents the amino acids after the 713th amino acid); **(C)** shows the schematic diagram of the three-dimensional structure of the mutant GLI2 protein.

Figure 5A illustrates the *GLI2* gene structure, based on mRNA transcript NM_001374353.1, which contains 14 exons; the mutation identified in this case is significant. A schematic representation of the GLI2 protein's three-dimensional structure is shown in Figures 5B,C, where the gray region in Figure 5B denotes amino acids beyond position 713 in the wild-type protein, and Figure 5C shows the truncated mutant form.

In a review of 112 individuals from 65 families carrying *GLI2* variants, only 37% of those with truncating mutations showed both pituitary abnormalities and polydactyly, whereas non-truncating variants were associated with isolated pituitary phenotypes in 92% of cases, reflecting incomplete penetrance (10, 16–18). To date, the ClinVar database lists 47 pathogenic and 18 likely pathogenic *GLI2* variants, with truncating mutations (frameshift and nonsense variants) comprising 53.8%. Such truncating alterations typically exert substantial effects on protein structure and function, severely compromising transcriptional regulatory activity and contributing to the development of CJS (10).

The clinical features observed in this case differed from previously reported presentations, which may be explained by the incomplete penetrance and substantial phenotypic heterogeneity associated with *GLI2* mutations, as well as the potential influence of specific mutation sites. Consistent with recognized features of the disorder, the child demonstrated postaxial polydactyly, growth retardation, and recurrent epileptic seizures. Whole-exome sequencing confirmed a heterozygous variant, c.2137(exon13)G>T/p.(E713,857) (NM_001374353), establishing a definitive diagnosis of CJS. To our knowledge, this particular variant has not been previously documented.

Previous studies have shown that CJS displays wide phenotypic variability both among unrelated individuals and within affected families, and no clear genotype–phenotype correlation has been identified (24). In this family, the child's mother also presented with bilateral hexadactyly and short stature, and sequencing confirmed that she carried the same heterozygous *GLI2* variant. Several other maternal relatives reportedly had polydactyly, further highlighting intrafamilial variability. The family history also included other adverse outcomes: the proband's older brother died at six months of age from congenital heart disease and pneumonia (with incomplete clinical data), and the maternal aunt experienced two unexplained miscarriages. These findings illustrate the broad clinical spectrum associated with *GLI2* mutations. Comprehensive segregation analysis across the maternal lineage, however, could not be completed due to practical constraints.

Existing research has not demonstrated a consistent correlation between specific *GLI2* variants and clinical severity, and considerable overlap exists in the phenotypic profiles of individuals carrying different mutations. Previous studies have also shown no association between the position or length of truncating variants and resulting phenotypes (10–12, 19, 21). One possible explanation is that the transcriptional activation domain of *GLI2* is located at the distal end of the protein; thus, truncating mutations typically eliminate this domain, leading to loss of activation function and resulting in similar functional consequences across most truncating variants. Furthermore, patients with microdeletions involving *GLI2* are more likely to present with additional congenital anomalies, including cardiac defects, visceral heterotaxy, and genitourinary malformations (5, 12).

Mutations in *GLI2* have been implicated in both Culler-Jones syndrome (CJS) and holoprosencephaly type 9 (HPE9). HPE9 encompasses a broad range of developmental brain abnormalities, which may occur with or without defects in forebrain cleavage. Because CJS and HPE9 share overlapping causative genes and clinical features, distinguishing between them is an important diagnostic challenge (10). HPE9 is considered part of the broader phenotypic spectrum of *GLI2*-related disorders and, like CJS, demonstrates incomplete penetrance and significant phenotypic variability. Increasing evidence suggests that CJS and HPE9 represent the milder and more severe ends of a continuous spectrum, respectively (5).

Most truncating *GLI2* variants reported to date are associated with polydactyly (8). In a large cohort study of 400 individuals with holoprosencephaly, Bear et al. identified 112 carriers of *GLI2* variants, including 43 with truncating mutations. Patients with truncating variants were significantly more likely to show the combination of pituitary abnormalities and postaxial polydactyly than those harboring variants of uncertain significance, suggesting that truncating *GLI2* mutations generally manifest as hypopituitarism, polydactyly, and subtle craniofacial features rather than classic holoprosencephaly. The presence of polydactyly in a patient with hypopituitarism or in family members should therefore prompt consideration of a *GLI2* mutation. Isolated holoprosencephaly caused by pathogenic *GLI2* variants appears to be extremely rare (10).

Beyond pituitary and limb defects, *GLI2*-related disorders have been linked to a range of systemic abnormalities, including renal anomalies (such as agenesis or dysplasia), urethral stenosis, congenital heart defects (e.g., atrial or ventricular septal defects), and neuroanatomical abnormalities including agenesis of the corpus callosum, periventricular venous system anomalies, and cortical gyration defects (2, 14, 25). According to existing literature, *GLI2* mutations commonly result in hypoglycemia, hypopituitarism, and abnormalities in neurological development (26, 27). Despite these established associations, functional characterization of the specific variant identified in this case remains lacking, as no animal model–based studies are available. More extensive basic research is needed to clarify the pathogenic mechanisms underlying this mutation's association with CJS.

In conclusion, this case reports a previously unrecognized truncating *GLI2* mutation in a neonate who presented with hallmark features of Culler-Jones syndrome. Given the substantial clinical variability and the absence of a clear genotype–phenotype correlation, CJS should be considered in infants who present with postaxial polydactyly, growth delay, refractory seizures, and a suggestive family history. Early genetic evaluation is strongly recommended to facilitate timely and accurate diagnosis.

## Data Availability

The datasets presented in this study can be found in online repositories. The names of the repository/repositories and accession number(s) can be found in the article/Supplementary Material.
